# Pegaptanib sodium for neovascular age-related macular degeneration: third-year safety results of the VEGF Inhibition Study in Ocular Neovascularisation (VISION) trial

**DOI:** 10.1136/bjo.2007.132597

**Published:** 2008-07-09

**Authors:** L J Singerman, H Masonson, M Patel, A P Adamis, R Buggage, E Cunningham, M Goldbaum, B Katz, D Guyer

**Affiliations:** 1 Retina Associates of Cleveland, Cleveland, Ohio, USA; 2 Ophthotech Corporation, Princeton, New Jersey, USA; 3 Optherion Inc, New Haven, Connecticut, USA; 4 Jerini Ophthalmic, New York, USA; 5 Pfizer, New York, New York, USA; 6 California Pacific Medical Center, San Francisco, California, USA; 7 Department of Ophthalmology, Stanford University School of Medicine, Stanford, California, USA; 8 Eyetech Inc, New York, New York, USA; 9 Danube Pharmaceutials Inc, New York, New York, USA; 10 SV Life Sciences, Boston, Massachusetts, USA

## Abstract

**Aims:**

To evaluate the safety of up to 3 years of pegaptanib sodium therapy in the treatment of neovascular age-related macular degeneration (NV-AMD).

**Methods:**

Two concurrent, prospective, multicentre, double-masked studies randomised subjects with all angiographic lesion compositions of NV-AMD to receive intravitreous pegaptanib sodium (0.3, 1 and 3 mg) or sham injections every 6 weeks for 54 weeks. Those initially assigned to pegaptanib were rerandomised to continue or discontinue therapy for 48 more weeks; sham-treated subjects continued sham, discontinued or received pegaptanib. At 102 weeks, subjects receiving pegaptanib 0.3 mg or 1 mg in years 1 or 2 continued; those receiving pegaptanib 3 mg or who did not receive treatment in years 1 and 2 were rerandomised to 0.3 mg or 1 mg for year 3.

**Results:**

As in years 1 and 2, pegaptanib was well tolerated in year 3. Adverse events were mainly ocular in nature, mild, transient and injection-related. Serious adverse events were rare. No evidence of systemic safety signals attributed to vascular endothelial growth factor inhibition arose in year 3. There were no findings in relation to vital signs or electrocardiogram results suggesting a relationship to pegaptanib treatment.

**Conclusion:**

The 3-year safety profile of pegaptanib sodium was favourable in patients with NV-AMD.

Vascular endothelial growth factor (VEGF) plays important roles in a wide variety of physiological processes, some reflecting its role in promoting angiogenesis and others important for normal physiological functions, such that issues of safety are of particular concern for long-term therapies premised on its inhibition. Physiological VEGF expression is now known to be important for protection of hepatocytes^1^ and renal cells,^2^ ^3^ for wound healing,^4^ ^5^ female reproductive cycling,^6^ ^7^ bone growth,^6^ ^8^ trophic maintenance of capillaries^9^ and neurons.^10^ In the eye, VEGF plays a physiological role in the development and trophic maintenance of the choriocapillaris^11^ ^12^ and in protecting retinal neurons from apoptosis in conditions of ischaemia.^13^ ^14^ The systemic use of bevacizumab (Avastin), a humanised antibody blocking all VEGF isoforms, in cancer treatment regimens has been accompanied by increased incidences of systemic hypertension, bleeding, proteinuria, gastrointestinal perforations and thromboembolic events.^15^^–^17 While the intravitreous mode of administration of anti-VEGF agents would be expected to reduce the severity of these systemic events, potential concerns remain, given the systemic levels, observed response in the fellow eye after intravitreous administration and the long-term maintenance therapy that some patients may require.^18^ ^19^

Preclinical findings in animal models have suggested that the selective inhibition by intravitreous pegaptanib, which targets VEGF_165_, an especially proinflammatory isoform,^20^^–^22 and spares VEGF_120_, could minimise some of the safety issues. While providing clinical benefit in the treatment of all angiographic subtypes of neovascular age-related macular degeneration (NV-AMD),^23^ ^24^ continuous intravitreous pegaptanib sodium has been shown to have an excellent safety profile over 2 years.^23^ ^25^ We now present further data for patients who have continued for 3 years in the VEGF Inhibition Study in Ocular Neovascularisation (VISION) trial, showing that no new safety signals have emerged over this additional period.

## MATERIALS AND METHODS

### Study design

**Figure 1:**
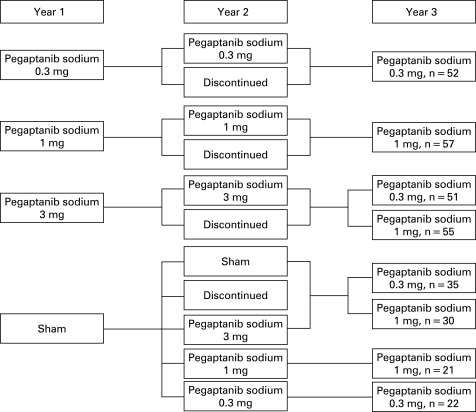
Randomisation at years 1, 2 and 3.

The details of the design of the VISION trial have been reported.^23^ ^25^ The study protocol was reviewed and approved by an institutional review board at each study site in accordance with the guidelines for the conduct of clinical research in the 1964 Declaration of Helsinki. Study participants provided signed informed consent before baseline procedures were performed. In brief, two identically designed, phase 3, prospective, comparative studies comprised the trial; both had randomised, double-masked, controlled, dose-ranging, multicentre, parallel-group designs (fig 1). At baseline, patients were randomised to one of four treatment groups (0.3 mg, 1 mg or 3 mg of pegaptanib sodium in 0.09 ml of solution or sham injections) and received nine intravitreous or sham injections once every 6 weeks for 48 weeks with follow-up to week 54. At week 54, subjects in the active therapy arms were rerandomised (1:1) to either discontinue or continue treatment for a further 48 weeks (eight injections). Subjects receiving sham were rerandomised at week 54 (1:1:1:1:1) to discontinue sham treatment, to continue on the study receiving one of the three active treatments or to continue on sham therapy.

At 102 weeks, 422 remaining subjects with visual acuity in the study eye of 20/800 or better continued treatment for a further nine injections in the third year (week 102 to week 150 with follow-up to week 156). Those receiving the 0.3 mg or 1 mg dose in year 2 continued on the same treatment, while subjects receiving the 3 mg dose or sham were rerandomised to 0.3 mg or 1 mg (1:1). Subjects not on treatment during the second year but who had received 0.3 mg or 1 mg during the first year resumed treatment at the previous dose. Subjects not on treatment during year 2 but who had received 3 mg or sham during year 1 were rerandomised to receive 0.3 mg or 1 mg (1:1). In year 3, study participants were masked to the actual dose being administered but were aware that only active 0.3 mg and 1 mg doses were being administered.

### Clinical monitoring

Throughout the study, safety was assessed by adverse event reporting, ophthalmic examination, applanation tonometry, laboratory assessments, electrocardiograms (ECGs) and vital signs. Laboratory tests included haematology (complete blood counts) and chemistry (electrolytes, renal and hepatic) assessments. Adverse events were assessed by investigators for the relationship to the injection procedure and to study the drug.

### Independent data monitoring

An Independent Data Monitoring Committee consisting of experts independent from the sponsors and the investigators was established before the trial’s start. The committee reviewed the data and procedures of both studies on an ongoing basis to ensure subject safety.

### End points and analyses

Safety end-points included all adverse events and serious adverse events (ophthalmic or systemic), intraocular pressure (IOP), clinical laboratory data, ECG abnormalities and changes, and loss of >20 letters (four lines; Early Treatment of Diabetic Retinopathy Study) of visual acuity between visits. Adverse events were coded according to the Medical Dictionary for Regulatory Activities 5.1. If the subject experienced more than one occurrence of the same event, only the event with the highest severity value was counted.

Safety data were tabulated for the combined population of the two studies, and year 3 adverse events were those with an onset date from week 102 (including the injection day at week 102) up to week 156 (excluding the injection day at week 156). Unless otherwise specified, the safety population included all subjects who received active pegaptanib sodium therapy at any dose throughout years 1, 2 and 3. Analyses of injection-related serious adverse events included all subjects who received pegaptanib sodium in year 3 regardless of previous randomised treatment (ie, sham or treatment discontinued). Assessments of IOP changes were performed in subjects who received 0.3 mg or 1 mg of pegaptanib sodium continuously from years 1 to 3. Statistical analyses on year 3 data were limited by small sample sizes and were considered to be descriptive.

## RESULTS

In all, 165 subjects who received active therapy with pegaptanib sodium in the first 2 years of the study were rerandomised at week 102. The rerandomised population was predominantly white with a higher ratio of females to males and a mean age in the mid-70s. Patient groups generally were similar to those reported at 54 and 102 weeks.^23^^–^25 The primary safety population consisted of the 161 subjects who received at least one dose of study medication in year 3 (75 received 0.3 mg, and 86 received 1 mg; four patients were randomised but did not receive study medication) and who received active treatment throughout years 1 and 2. The total number of injections administered in these 161 subjects during year 3 was 1254 (mean injections per patient = 7.8). In all, 422 subjects received a total of 3227 injections of pegaptanib sodium 0.3 mg or 1 mg in year 3 regardless of treatment in previous years.

As seen in years 1 and 2, pegaptanib sodium was well tolerated in year 3, and adverse events were mainly ocular in nature, mild and predictable. Ocular adverse events occurred in 114/161 (71%) subjects in the primary safety population in year 3, and the majority of events were associated with the injection procedure (table 1). Twenty-seven (17%) subjects experienced serious ocular or non-ocular adverse events, and 3% discontinued due to an adverse event.

**Table 1:** Adverse event summary, study eye (year 3 safety population) (n (%))

	All subjects who received active therapy for 3 years n = 161
Subjects with any adverse event	142 (88)
Subjects with an ocular adverse event	114 (71)
Injection-procedure related	84 (52)
Study-therapy related	19 (12)
Subjects with any serious adverse event	27 (17)
Subjects discontinuing due to an adverse event	5 (3)

Ocular adverse events reported in study eyes in ⩾5% of the primary safety population subjects are summarised in table 2. The most common ocular adverse events were punctate keratitis (41/161 (25%)), increased IOP (32/161 (20%)), eye pain (27/161 (17%)), and cataract (23/161 (14%)). The incidence of these events was similar to or less than the incidence in years 1 and 2 of the study. As in previous years, no severe anterior chamber inflammation was seen, and the assessment of cataract in phakic subjects did not suggest that pegaptanib sodium injections were associated with cataract progression. During the third year, 8/161 (5%) subjects lost 20 or more letters of vision, and five of these fully or partially improved in subsequent visits.

**Table 2:** Adverse events reported in study eyes in ⩾5% of subjects (year 3, safety population) (n (%))

Adverse event	All subjects who received active therapy for 3 years n = 161
Punctate keratitis	41 (25)
Intraocular pressure increased	32 (20)
Eye pain	27 (17)
Cataract	23 (14)
Vitreous floaters	18 (11)
Conjunctival haemorrhage	15 (9)
Anterior chamber inflammation	14 (9)
Corneal oedema	13 (8)
Vitreous opacities	13 (8)
Visual acuity reduced	10 (6)
Eye pruritus	8 (5)
Lacrimation increased	8 (5)

Injection-related serious adverse events were rare in the 422 subjects treated with pegaptanib sodium 0.3 mg or 1 mg in year 3, regardless of treatment in previous years (table 3). Two cases of endophthalmitis (0.06% per injection, 0.47% per patient), one case of rhegmatogenous retinal detachment (0.03% per injection, 0.24% per patient) and no cases of traumatic cataract were reported. One case of vitreous haemorrhage was reported as a serious ocular adverse event and was assessed by the investigator to be related to the injection procedure. One reported event of reduced visual acuity of at least four lines between two consecutive visits (experienced by a subject who also had experienced endophthalmitis) was assessed to be related to the injection procedure. In addition, one subject experienced retinal haemorrhage and vitreous haemorrhage that were assessed to be related to the study drug, but not reported as a serious ocular adverse event.

**Table 3:** Serious ocular adverse events reported in study eyes (year 3) (number of cases (rate per injection))

Adverse event	All subjects who received pegaptanib sodium 0.3 mg or 1 mg in year 3 n = 422 (no of injections = 3227)
Endophthalmitis	2 (0.06% per injection)
Rhegmatogenous retinal detachment	1 (0.03% per injection)
Traumatic cataract	0 (0.0% per injection)
Vitreous haemorrhage	1 (0.03% per injection)

The general pattern of IOP changes seen in years 1 and 2 continued in year 3. On average, subjects experienced an increase in IOP at the 30 min postinjection assessment compared with preinjection at each treatment visit. At the 1-week postinjection assessment, the pressure had returned to levels similar to preinjection. Of subjects treated with 0.3 mg or 1 mg of pegaptanib sodium continuously for 3 years, the mean IOP remained stable throughout the 3 years, and more than 80% of subjects did not experience IOP values ⩾35 mm Hg at any time. Intervention for increased IOP on injection days also was relatively infrequent. Ten of 109 (9%) underwent paracenteses, and only 17 (16%) required treatment with concomitant medications for increased IOP on one or more injection days during the 3-year follow-up.

The Independent Reading Center graded all baseline and week 30, 54, 78, 102 and 156 fluorescein angiograms. These examinations revealed no retinal vascular abnormalities that are unexpected in the natural history of NV-AMD. Specifically, there were no notable delays in arteriovenous transit time, or abnormalities in choroidal perfusion or arteriolar occlusions.

As in years 1 and 2, no evidence of systemic safety signals related to the inhibition of VEGF arose with the third year of administration of pegaptanib sodium. In the 161 subjects receiving active therapy for 3 years, the most common nonocular adverse events in year 3 were infections and infestations (18%); respiratory, thoracic and mediastinal disorders (15%); and gastrointestinal disorders (14%) (table 4). There were no thromboembolic cerebrovascular accidents; only two subjects had an event of myocardial infarction (2%), and one subject had an event of angina (1%). The incidence of these thromboembolic events was the same among the entire cohort of 422 subjects treated with pegaptanib sodium in year 3 and was similar to those observed in the pegaptanib and sham groups in years 1 and 2. There were no serious nonocular haemorrhagic events. Among the entire cohort of 422 subjects treated with pegaptanib sodium in year 3, the most frequently occurring serious adverse events were neoplasms and cardiac disorders, each experienced by 12 subjects (3%); gastrointestinal disorders (10 subjects, 2%); and vascular disorders (nine subjects, 2%). With the exception of one event of hypertension that the investigator assessed to be related to the injection procedure (the event occurred before an injection, and the investigator considered that the event was due to the subject’s emotions in connection with the subsequent injection), none of these events was related to the injection procedure or the study drug. There were six deaths (1%) among the 422 subjects who received pegaptanib sodium in year 3; none of the deaths was considered to be related to study drug or injection procedure by the investigator. The adverse events associated with these death cases were glioblastoma, cardiac arrest, *Clostridium* colitis, cardiorespiratory arrest, hypotension and metastatic lung cancer, one case each.

**Table 4:** All-causality non-ocular adverse events in ⩾5% of subjects (year 3, safety population) (n (%))

Adverse event (system organ class)	All subjects who received active therapy for 3 years n = 161
Investigations*	41 (25)
Infections and infestations	29 (18)
Respiratory, thoracic and mediastinal disorders	24 (15)
Gastrointestinal disorders	23 (14)
Musculoskeletal and connective tissue disorders	20 (12)
Nervous system disorders	18 (11)
Cardiac disorders	17 (11)
Injury, poisoning and procedural complications	16 (10)
Vascular disorders	15 (9)
Skin and subcutaneous tissue disorders	14 (9)
Metabolism and nutrition disorders	10 (6)
Neoplasms benign, malignant and unspecified	9 (6)

*The majority of these events were “increased intraocular pressure (IOP)”; the investigators were required to report an IOP of ⩾30 mm Hg at 30 min after injection as an adverse event.

**Figure 2:**
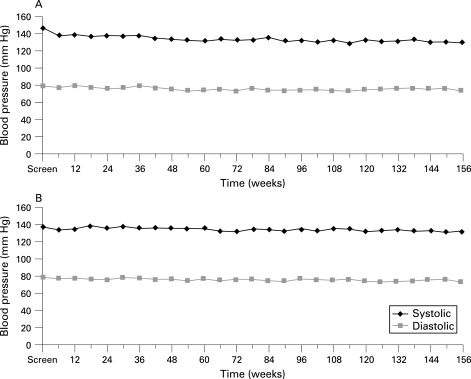
(A) Mean blood pressure through 3 years: subjects who received 0.3 mg of pegaptanib sodium for all 3 years, n = 52. (B) Mean blood pressure through 3 years: subjects who received 1 mg of pegaptanib sodium for all 3 years, n = 57.

There were no findings in relation to vital signs performed at each clinical assessment or ECG test results that were suggestive of a relationship to treatment with pegaptanib sodium; in particular, there was no evidence of an increase in mean blood pressure over the 3 years of treatment (fig 2). There were no laboratory test findings suggestive of a relationship with pegaptanib sodium. Specifically, when the median change from baseline was reviewed for the laboratory parameters, there was no evidence of a clinically meaningful pattern. Additionally, analysis of clinically significant laboratory abnormalities for individual patients revealed the majority to be transient, with no findings to suggest a relationship between treatment with pegaptanib sodium and these abnormalities.

## DISCUSSION

The data from the VISION trial demonstrate a favourable ocular and systemic safety profile for 3 years of continuous selective VEGF inhibition with pegaptanib sodium for the treatment of NV-AMD. No new safety signals emerged during the third year of therapy with pegaptanib sodium. Subjects’ compliance with intravitreal injections of pegaptanib sodium during the third year also was consistent with the previous 2 years of the study.

There was no change in the ocular safety profile of pegaptanib sodium in the third year. In year 3, as in the previous years, adverse events were mainly ocular in nature, mild, transient and related to the injection procedure. The incidence of the most commonly reported ophthalmic adverse events (eg, punctate keratitis, eye pain, vitreous floaters and cataract) decreased during the second^25^ and third years of the studies. While there was no sham group in the third year of the study, the most common ocular events in years 1 and 2 were reported in a higher proportion of study eyes of sham patients relative to the fellow eyes of each active treatment group, suggesting that many of these events may be related to the preparation procedure for the intravitreous injection rather than to the intravitreous injection itself. There was no case of severe anterior chamber inflammation, which suggests that no significant intraocular activation of the immunological response was triggered. This was consistent with previous studies that employed an aptamer for therapy^26^ and with those demonstrating an absence of detectable serum antibodies against pegaptanib.^27^ Although extremely rare cases of anaphylactoid reactions have been reported in patients receiving pegaptanib (Macugen (package insert). New York: (OSI) Eyetech; 2006), it is not clear if the response is triggered by pegaptanib or some other component of the preparation or procedure. Additionally, there was no evidence of a difference in cataract progression between the third year and previous years. No unexpected changes in the choroidal and retinal vasculatures were seen even after 3 years of selective VEGF inhibition.

The low rate of serious ocular events continued during study year 3, with the incidence of rhegmatogenous retinal detachment only 0.03% per injection (1/3227), and no iatrogenic traumatic cataracts were reported. The rate of endophthalmitis continued to decline over the course of the studies from 0.16% per injection (12 of 7545 injections) in the first year to 0.10% per injection (4/4091) in the second year^25^ and to 0.06% per injection (2/3227) in the third year (p<0.0001, Cochran–Armitage trend test). This decrease in the rate of endophthalmitis may result at least partially from a protocol amendment emphasising the importance of adherence to aseptic technique when performing intravitreal injections. The lower rate of endophthalmitis also may be attributed to the widespread adoption of and increasing familiarity with intravitreous injections by retina specialists over the 3 years of this study.

There was also no change in the systemic safety profile of pegaptanib sodium. The types and incidence of systemic serious adverse events observed are not unexpected in this elderly patient population, and none of these events was judged to be related to study drug. This favourable systemic safety profile is of particular importance for the NV-AMD population that is at higher risk for cardiovascular and thromboembolic diseases.^28^ In conclusion, 3 years of continuous selective VEGF inhibition with pegaptanib sodium in the treatment of patients with NV-AMD confirmed the favourable safety profile of pegaptanib sodium.

## Appendix

The following members of the Writing Committee and Independent Data Management and Safety Monitoring Committee participated in the VEGF Inhibition Study in Ocular Neovascularisation (VISION) Clinical Trial Group study.

### Writing Committee

LJS, Cleveland, OH (chair and corresponding author), HNM, MP, APA, RB, ETC, MG, BK, DRG.

### Independent Data Management and Safety Monitoring Committee

A Bird, Moorfields Eye Hospital (chair); D D’Amico, Massachusetts Eye and Ear Infirmary (chair emeritus); J Herson, Johns Hopkins University; R Klein, University of Wisconsin.

A list of the members of the Steering Committee, Data Management and Statistics Group, Eligibility and Classification Quality Assurance Team, Independent Fundus Photography and Angiogram Reading Center, the VISION Clinical Trial Group, and (OSI) Eyetech and Pfizer Staff who participated in the VEGF Inhibition Study in Ocular Neovascularisation (VISION) trial is available at http://aaojournal.org

### Supplemental material for website

#### Steering Committee

M Blumenkranz, Stanford University; M Buyse, International Drug Development Institute; M Goldberg, Johns Hopkins University; ES Gragoudas, J Miller, Massachusetts Eye and Ear Infirmary; SD Schwartz, University of California at Los Angeles; L Singerman, Retina Associates of Cleveland; L Yannuzzi, Columbia University; AP Adamis, DR Guyer, D O’Shaughnessy, (OSI) Eyetech, Inc.

#### Data Management and Statistics Group

M Buyse, S de Gronckel, G Fesneau, E Quinaux, D Tremolet, K Wang, International Drug Development Institute, Brussels and Boston; A Brailey, J Finman, N Ting, Pfizer Inc, Groton, Connecticut.

#### Eligibility and Classification Quality Assurance Team

NM Bressler, SB Bressler, R Denblow, OD Schein, S Seabrook, S Solomon, AP Schachat, D Philips, Wilmer Ophthalmological Institute, Johns Hopkins University.

#### Independent Fundus Photograph and Angiogram Reading Center

M Altaweel, MD Davis, BA Blodi, RP Danis, MS Ip, C Hiner, J Elledge, M Webster, C Hannan, J Ficken, S Alexander, M Neider, H Wabers, P Vargo, E Lambert, L Kastorff, A Carr, A Shkiele, J Baliker, University of Wisconsin, Madison.

#### VISION Clinical Trial Group

R Guymer, Centre for Eye Research Australia, University of Melbourne, Melbourne, Australia; I Constable, Lions Eye Institute, Nedlands, Australia; J Arnold, S Sarks, Marsden Eye Specialists, Parramatta, Australia; A Chang, Eye and Vision Research Institute, Sydney, Australia; M Gillies, Save Sight Institute, Sydney, Australia; P Mitchell, Westmead Hospital, Westmead, Australia; A Haas, Universitäts Augenklinik, Graz, Austria; M Stur, Universitäts-Augenklinik, Vienna, Austria; A Leys, UZ St Rafaël, Leuven, Belgium; C Moreira, E Portella, Hospital de Olhos do Paraná, Curitiba, Brazil; M de Avila, AC Taleb, Universidade Federal de Goiás, Goiânia, Brazil; J Lavinsky, D Lavinsky, Hospital das Clínical de Porto Alegre, Porto Alegre, Brazil; ME Farah, Universidade Federal de São Paulo, São Paulo, Brazil; G Williams, Calgary Retina Consultants, Calgary, Alberta, Canada; B Leonard, University of Ottawa Eye Institute, Ottawa, Canada; R Garcia, Eye Centre Pasqua Hospital, Regina, Saskatchewan, Canada; D Maberley, Vancouver Hospital and Health Sciences Center, University of British Columbia Eye Care Center, Vancouver, British Columbia, Canada; JM Lopez, Pontificia Universidad Católica de Chile, Santiago, Chile; FJ Rodriguez, Fundación Oftalmológica Nacional, Bogotá, Colombia; I Fiser, Vinohrady Teaching Hospital, Prague, Czech Republic; M Larsen, Herlev Hospital, University of Copenhagen, Copenhagen, Denmark; J-F Korobelnik, Groupe Hospitalier Pellegrin, Bordeaux, France; G Soubrane, Centre Hospitalier Universitaire de Créteil-Paris XII, Créteil, France; F Koenig, Centre de Recherche en Ophtalmologie, Lyon, France; A Gaudric, Hopital Lariboisiere, Paris, France; S Dithmar, FG Holz, University of Heidelberg, Heidelberg, Germany; A Joussen, B Kirchhof, University of Cologne, Cologne, Germany; P Wiedemann, Universitätsklinikum Leipzig Klinik und Poliklinik, Leipzig, Germany; D Pauleikhoff, St Franziskus Hospital, Muenster, Germany; U Schneider, University Eye Clinic Tübingen, Tübingen, Germany; I Suveges, Semmelweis University, Budapest, Hungary; J Gyory, Csolnoky Ferenc County Hospital, Kórház, Hungary; A Pollack, Kaplan Medical Center, Rehovot, Israel; A Loewenstein, Ichilov Medical Center, Tel Aviv, Israel; I Rosenblatt, Rabin Medical Center, Petach-Tikva, Israel; A Giovannini, Istituto di Scienze Oftalmologiche, Ancona, Italy; U Menchini, II Clinica Oculistica Universitá degli Studi di Firenze, Florence, Italy; R Brancato, Università Ospedale San Raffaele, Milan, Italy; F Cardillo Piccolino, FM Grignolo, Università di Torino, Turin, Italy; F Bandello, Policlinico Universitario, Udine, Italy; RO Schlingemann, Academic Medical Center, University of Amsterdam, The Netherlands; A Deutman, UMC St Radboud, Institute of Ophthalmology, Nijmegen, The Netherlands; J Kaluzny, Akademia Medyczna ul M Sklodowskiej-Curie, Bydgoszcz, Poland; K Pecold, Akademia Medyczna ul Dluga, Poznán, Poland; J Cunha-Vaz, R da Silva, Associacão Para Investigacão Biomedica e Inovacão Em Luz e Imagem, Universidada de Coimbra, Coimbra, Portugal; JM Ruiz Moreno, VISSUM-Instituto Oftalmologico de Alicante, Alicante, Spain; J Mones, Instituto de Microcirugia Ocular, Barcelona, Spain; M Figueroa, Hospital Oftalmologico Internacional, Madrid, Spain; C Pournaras, Hopital Cantonal de Geneva, Geneva, Switzerland; L Zografos, Hopital Opthalmique Jules Gonin, Lausanne, Switzerland; N Lois, Aberdeen Royal Infirmary, Aberdeen, UK; U Chakravarthy, Queen’s University Belfast Royal Victoria Hospital, Belfast, UK; P Hykin, Moorfields Eye Hospital, London, UK; I Chisholm, Southampton General Hospital, Southampton, UK; MW Johnson, WK Kellogg Eye Center, Ann Arbor, Michigan; DM Marcus, Medical College of Georgia, Augusta, Georgia; N Mandava, University of Colorado Rocky Mountain Lions Eye Institute, Aurora, Colorado; JA Haller, Wilmer Ophthalmological Institute, Johns Hopkins University, Baltimore, Maryland; F Cangemi, Vitreo-Retinal Associates of New Jersey, Belleville, New Jersey; D Boyer, Retina Vitreous Associates, Beverly Hills, California; I Kim, J Loewenstein, Massachusetts Eye and Ear Infirmary, Boston, Massachusetts; J Heier, Ophthalmic Consultants of Boston, Boston, Massachusetts; E Reichel, New England Eye Center, Boston, Massachusetts; PM Falcone, Connecticut Retina Consultants, Bridgeport, Connecticut; DJ Weissgold, University of Vermont College of Medicine, Burlington, Vermont; BP Conway, University of Virginia, Charlottesville, Virginia; R Garfinkel, Retina Group of Washington, Chevy Chase, Maryland, B Glaser, Glaser Murphy Retina Treatment Centers, Chevy Chase, Maryland; AT Lyon, Northwestern University Sorrel Rosin Eye Center, Chicago, Illinois; H Lewis, Cleveland Clinic Cole Eye Institute, Cleveland, Ohio; JA Wells, Palmetto Retina Center, Columbia, South Carolina; L Wilcox, Eye Center of Concord, Concord, New Hampshire; G Fish, Texas Retina Associates, Dallas, Texas; D Eliott, Kresge Eye Institute, Detroit, Michigan; S Fekrat, Duke University Eye Center, Durham, North Carolina; B Taney, Retina Vitreous Consultants, Fort Lauderdale, Florida; AM Eaton, Retina Health Center, Fort Myers, Florida; V Deramo, LI Vitreo-Retinal Consultants, Great Neck, New York; J Wroblewski, Cumberland Valley Retina Center, Hagerstown, Maryland; D Tom, New England Retina Associates, Hamden, Connecticut; DR Chow, DH Orth, KH Packo, Illinois Retina Associates, Harvey, Illinois; E Holz, W Mieler, Baylor College of Medicine, Houston, Texas; B Kuppermann, University of California, Irvine, California; N Sabates, Eye Foundation of Kansas City, Kansas City, Missouri; H Cummings, Southeastern Retina Associates, Knoxville, Tennessee; SD Pendergast, Retina Associates of Cleveland, Cleveland, Ohio; C Gonzales, Jules Stein Eye Institute, Los Angeles, California; JI Lim, Doheny Eye Institute, University of Southern California, Keck School of Medicine, Los Angeles, California; S Charles, Charles Retina Institute, Memphis, Tennessee; S Sanislo, Stanford University School of Medicine, Menlo Park, California; P Rosenfeld, Bascom Palmer Eye Institute, Miami, Florida; T Connor, Eye Institute, Milwaukee, Wisconsin; H Cantrill, VitreoRetinal Surgery, Minneapolis, Minnesota; R Willson, Foundation for Retinal Research, New Orleans, Louisiana; K Bailey-Freund, Manhattan Eye, Ear & Throat Retinal Research Office, New York; R Rosen, New York Eye and Ear Infirmary, New York; R Leonard, Dean A McGee Eye Institute, Oklahoma City, Oklahoma; A Brucker, Scheie Eye Institute, Philadelphia, Pennsylvania; A Ho, Wills Eye Hospital Retina Research, Philadelphia, Pennsylvania; S Sneed, Retinal Consultants of Arizona, Phoenix, Arizona; T Friberg, UPMC Eye Center, Pittsburgh, Pennsylvania; M Klein, Casey Eye Institute, Portland, Oregon; P Tornambe, Retina Consultants, Poway, California; G Stoller, Ophthalmic Consultants of Long Island, Rockville Centre, New York; A Capone, Jr, Associated Retinal Consultants, Royal Oak, Michigan; PS Bernstein, John Moran Eye Center, University of Utah, Salt Lake City, Utah; HR McDonald, H Schatz, RN Johnson, West Coast Retina Medical Group, San Francisco, California; M Nanda, Orange County Retina, Santa Ana, California; R Avery, California Retina Consultants, Santa Barbara, California; K Wong, Sarasota Retina Institute, Sarasota, Florida; WS Grizzard, Retina Associates of Florida, Tampa, Florida; P Higgins, Retina Associates of New Jersey, Teaneck, New Jersey; H Hudson, Retina Centers PC Tucson, Arizona; L Joffe, Retina Associates SW PC, Tucson, Arizona; M Varenhorst, Vitreo-Retinal Consultants and Surgeons, Wichita, Kansas; MM Slusher, Wake Forest University Eye Center, Winston-Salem, North Carolina.

#### (OSI) Eyetech and Pfizer staff (at the time the study was conducted)

AP Adamis, F Betts, E Cunningham, Jr, K Curtiss, DR Guyer, E Harrison, B Katz, HN Masonson, R DeMarco, D O’Shaughnessy, (OSI) Eyetech Inc, New York; C Beals, M Patel, I Rodriguez, Pfizer Inc, New York; C Starita, Pfizer Ltd, UK.
